# Data management and sharing policy: the first step towards promoting data sharing

**DOI:** 10.1186/s12916-019-1315-8

**Published:** 2019-04-17

**Authors:** Naomi Waithira, Brian Mutinda, Phaik Yeong Cheah

**Affiliations:** 1 Mahidol Oxford Tropical Medicine Research Unit (MORU), Faculty of Tropical Medicine, Mahidol University, 420/6 Rajvithi Road, Bangkok, 10040 Thailand; 2 Centre for Tropical Medicine and Global Health, Nuffield Department of Clinical Medicine, University of Oxford, Old Road Campus, Roosevelt Drive, Oxford, OX3 7FZ UK; 3 The Ethox Centre, Nuffield Department of Population Health, University of Oxford, Old Road Campus, Roosevelt Drive, Oxford, OX3 7FZ UK

**Keywords:** Data sharing, Data management, Data sharing policy, Broad consent, Ethical

## Abstract

**Background:**

Health-related research funders, regulators and journals expect that de-identified individual-level health data be shared widely, with as few restrictions as possible; yet, in reality, the volume of shared data remains low.

**Main body:**

Health researchers and other data producers are reluctant to share their data unless they are confident that their datasets are of high quality and reliable, and that they are used in accordance with the values and aims of their institutions. We argue that having an institutional, departmental or group data management and sharing policy is the first step towards encouraging researchers and healthcare professionals to share their data more widely. Our paper outlines the elements of a data management and sharing policy, which should include aims consistent with those of the institution as well as with data management procedures, models of data sharing, request procedures, consent models and cost recovery mechanisms. A policy would help an institution, department or group maximise the use of its data and protect the interests of the institution and its members. We base our recommendations on our experience collecting and curating data for large clinical trials conducted in low- and middle-income countries, facilitating the sharing of datasets with secondary users, whilst teaching data management and conducting empirical research on data sharing. Although the fundamentals of a policy are general, the paper is focused on the low- and middle-income country context.

**Conclusion:**

We argue that having an institutional, departmental or group data management and sharing policy is the first step in promoting data sharing.

## Background

Health-related research funders, regulators and journals are increasingly expecting that individual-level health data will be shared more widely [1–3]; yet, in reality, the volume of data shared remains low [4]. Rationales for sharing data include maximising the utility of datasets and improving the rigour and transparency of research with the ultimate aim of improving health [5–8]. Sharing health data from all settings has potential benefits; however, for data collected in low- and middle-income countries (LMICs), it is critical due to the disproportionately smaller number of studies conducted or data collected in LMICs compared to the disease burden in these countries, making the sharing of datasets from LMIC settings particularly valuable to maximising the use of data.

We argue that a prerequisite to data sharing is to have a data management and sharing policy as well as associated processes, tools and governance mechanism in place [9]. We acknowledge that data sharing is indeed occurring, albeit without the existence of institutional policies and with gaps such as inequity in data access and reuse. In addition, much data sharing occurs without the implementation of basic data management standards, e.g. the sending of datasets via non-secure channels such as email. A policy would help an institution, department or research group generate high quality data, maximise the use of its data and gain better control over its data assets.

Funders and journals set their requirements based on their specific goals and interests; most health research funders expect that research data should be made openly available with as few restrictions as possible, while journals require data to be made transparently available for the purpose of validating the publication, yet not necessarily openly available [3]. Subtle discrepancies in the requirements from funders and journals mean that neither is all-inclusive. Institutional policies should be specific to the research context and exist to promote the interests of the institution as well as to safeguard the interests of their researchers and study participants. Institutional policies should not replace other policies but rather complement them and provide direction for researchers when there are discrepancies between the funder and journal requirements.

However, not many groups, particularly in LMICs, have a data sharing policy [4]. Institutions may not be well informed on what sharing options are available and what needs to be in place in order to share their data [10]. Furthermore, very few researchers in LMICs have requested data for secondary analyses; this has been attributed to poor digital literacy and data science skills as well as a lack of funding and time dedicated to research [4]. Our experience illustrates this – the Mahidol Oxford Tropical Medicine Research Unit (MORU) Data Access Committee (DAC) has received more than 30 applications since its establishment in 2016, yet nearly all requests have come from high-income countries (HICs) [11, 12]. This illustrates what LMIC researchers fear, namely that data sharing benefits HIC researchers and disadvantages LMIC researchers, thereby exacerbating existing inequalities between researchers in these settings [13].

Given the requirements of data sharing by funders and journals [1–3], having a data management and sharing policy places an institution in a stronger position when applying for funding and when submitting journal papers for publication. Institutions that have had the experience of sharing datasets may also be motivated to apply for datasets for secondary use, which in turn could increase their research output. Other potential incentives for data sharing are the increase of collaborations, visibility, citations, impact, and funding opportunities [4].

Our paper outlines the elements of a data management and sharing policy. We base our recommendations on our experience collecting and curating data for large clinical trials conducted in LMICs [14, 15], facilitating the sharing of datasets with secondary users [11], teaching data management and conducting empirical research on data sharing. Although the fundamentals of a policy are applicable to all health-related institutions, our paper is focused on the LMIC context. We recognise that LMIC environments differ markedly from HICs in terms of level of funding, data management capacity, IT and basic facilities such as power and internet connectivity [4, 10].

## Elements of a data management and sharing policy

### Aims

A data management and sharing policy should be consistent with institutional aims; for example, an institution with the aim to improve the treatment of malaria should have a policy that supports the sharing of data that ultimately contributes to malaria treatment improvement. These aligned aims help researchers make data sharing plans that maximise the use of their data in both primary and secondary analyses, and could serve as a powerful internal incentive. The policy should be specific to an institution’s area of work and in harmony with applicable ethics guidelines and regulatory requirements to ensure that researchers and research participants are protected from any potential harms. Therefore, in-depth consultations and engagement with key internal and external stakeholders may be necessary [8]. Scoping and cataloguing what type of data the institution collects (e.g. routinely collected health data or research data), supplemented by a review of funders’ and journal policies as well as local ethics and regulatory requirements, is key.

### Data management for data sharing

One of the basic requirements of effective data sharing is assurance that the data and reported results are credible and accurate, and that the rights, integrity and confidentiality of research participants are protected [16, 17]. Good data management ensures that high quality and credible datasets are produced. Data management refers to activities undertaken to organise and handle data throughout the study period – from study design and data collection, through to the dissemination of results, data sharing and archiving.

The data management and sharing policy should outline institutional requirements with regards to processes for collection, curation, storage and sharing of data, as well as provision of guidance related to study-specific data management and sharing plans. A study-specific data management and sharing plan details the procedures for a specific study, and is required by most biomedical research funders [18]. Consideration of data sharing at the outset of a research project ensures that sufficient resources are allocated to data management activities; this entails quantifying costs of items such as computer hardware, software, staff, personnel training and data archiving. The policy should also include the institutional requirements for data repositories as well as how to select suitable repositories. In addition, the policy should address the requirements for metadata such as the study protocol, annotated study case report forms [19], case report form completion guidelines and the data dictionary.

To implement data management for sharing, institutions should invest in building data management capacity through training of researchers and data support personnel, as well as through acquiring the necessary IT infrastructure, including servers and networks. Research teams should be aware of how electronic datasets are processed and converted into shareable datasets, and how datasets can be stored securely during the study period and beyond. This requires knowledge of principles of data coding and data formats, knowledge of methods for securing data from malicious harm, unintentional harm and from disasters, an understanding of methods for deidentification of data, and knowledge of available data management tools and software.

### Models of sharing

A data sharing policy should consider the different models of making data available to secondary users, including (1) online open access, e.g. as supplementary files to a journal article (with this method of sharing there is no oversight or control of secondary uses of the data); (2) external repository without case-by-case assessment such as Figshare (https://figshare.com/) (with this method, datasets submitted to a repository may be accessed by registered users who have agreed to the repository’s terms and conditions of use, and users of the data will be restricted by the terms and conditions of the repository); and (3) managed access via application to a DAC, a committee who have the responsibility of reviewing and assessing access requests, and subsequently approving or disapproving them. There are different types of DACs, including institutional DACs (e.g. the MORU DAC), independent DACs (e.g. the Clinical Study Data Request DAC, https://www.clinicalstudydatarequest.com/Default.aspx), and DACs specific to research consortiums.

Models of sharing will depend on certain considerations such as what type of datasets are collected, applicable regulations and consent models used. For a managed access model, the roles, responsibilities and membership of DACs should be defined. An institution should decide on the mechanisms for an application procedure and criteria for review of applications, including what the data will be used for, who is applying, any foreseen benefit of sharing and any potential harm to participants, primary researchers or their institutions.

### Data access criteria

The data sharing policy should provide guidelines, within constraints of funders’ and regulatory requirements, on when specific conditions of access should be put in place; this could include recognition requirements such as authorships, acknowledgements or standard citations. In some cases, collaborations may be necessary, especially where interpretation of the data requires the experience of the primary researchers and an in-depth understanding of the context. In addition, an institution may have exclusive access periods, requirements for benefit sharing, preferential access provisions (e.g. to LMIC researchers and collaborators) and embargo periods. In addition, the policy should mandate when formal data access agreements should be signed and who should be signatories to those agreements.

### Consent models for participants

Additionally, the different types of consent models, i.e. no consent, specific consent and broad consent, should also be considered [20]. There are merits and disadvantages as well as varying legality issues for each consent model and how these can be put into practice. ‘Broad consent’ has been widely proposed as a mechanism to enable potential research participants to give permission for their data to be used in future research studies with some restrictions [20, 21]. However, from our experience and that of others conducting studies in LMICs, participants rarely fully comprehend the information in primary studies [22–24]. Our recent empirical study showed that providing information on data sharing and obtaining broad consent for data sharing in addition to the consent for the primary study adds another layer of complexity to the consent process [25].

For a chosen consent model, there are requirements in the protocol, consent form, ethics application documents and training required for staff obtaining consent. For multicentre studies, it is necessary to engage with collaborators to ensure that clinical study agreements include provisions for data sharing and consent.

### Budgeting and cost recovery

Typically, data sharing is not designed to generate profit for primary researchers, but rather to share the actual costs between secondary users and primary researchers. The costs to be taken into consideration may include time spent on an activity specific to data sharing, such as curating data for sharing, as well as time spent by DAC members in reviewing data requests or by institutional lawyers where the shared data has legal implications, and general administrative expenses. Costs that could be shared between the primary and secondary users may include hardware, software, data storage and staff costs (including training), particularly for legacy datasets, where costs of preparation and sharing of data were not included in the initial grant proposals. The data sharing policy should include a description of data charges, if any, and how these are calculated.

## Conclusions

Herein, we outlined the elements of a data management and sharing policy for responsible data sharing [26]. We argue that having a policy is the first step to encourage researchers and other data producers to share their data. Primary researchers and data producers would be reluctant to share their data unless they are confident that their datasets are of good quality, collected and managed in accordance with accepted ethical and quality standards, and that the datasets are used in accordance with their values and aims as well as those of their institutions.

## Abbreviations

DAC: Data Access Committee
HIC: High-income country
LMIC: Low- and middle-income country
MORU: Mahidol Oxford Tropical Medicine Research Unit
